# Endocrine-metabolic imbalance drives osteoarthritis: From whole-joint pathobiology to precision therapy (Review)

**DOI:** 10.3892/ijmm.2026.5923

**Published:** 2026-07-09

**Authors:** Ruhui Yang, Haimin Zeng, Qi Xiao, Yining Xie, Yi Long, Xiang Chen

**Affiliations:** 1 Department of Rehabilitation Medicine, The Second Affiliated Hospital, Jiangxi Medical College, Nanchang University, Nanchang, Jiangxi 330006, P.R. China; 2 The Second Clinical Medical College, Jiangxi Medical College, Nanchang University, Nanchang, Jiangxi 330006, P.R. China; 3 The First Clinical Medical College, Jiangxi Medical College, Nanchang University, Nanchang, Jiangxi 330006, P.R. China

**Keywords:** osteoarthritis, endocrine-metabolic crosstalk, hormonal regulation, metabolic dysfunction, joint microenvironment, precision medicine

## Abstract

Osteoarthritis (OA) is a chronic degenerative joint disease closely associated with aging and metabolic dysfunction, characterized by cartilage degeneration, synovial inflammation, aberrant subchondral bone remodeling, pain and progressive functional impairment. Beyond mechanical loading, accumulating evidence indicates that OA is increasingly recognized as a whole-joint disorder shaped by the interplay between local tissue damage and systemic endocrine-metabolic imbalance. Endocrine factors, including sex hormones, thyroid hormone, melatonin, parathyroid hormone and vitamin D, together with metabolic disturbances, such as obesity, insulin resistance, dysregulated glucose and lipid metabolism and gut microbiota imbalances, can cooperatively remodel the joint microenvironment. Mechanistically, these alterations converge on immuno-inflammatory amplification, mitochondrial dysfunction, oxidative stress, cellular senescence, metabolic reprogramming and regulated cell death, thereby promoting extracellular matrix degradation, persistent synovitis and uncoupled bone-cartilage remodeling. The present review systematically summarizes the molecular basis of endocrine-metabolic crosstalk in OA and discusses emerging therapeutic opportunities targeting hormonal signaling, metabolic pathways, circadian regulation, nutritional support and lifestyle interventions. Nevertheless, the reciprocal interactions among endocrine signals, systemic metabolic abnormalities and local joint pathology remain incompletely understood, and their translation into mechanism-based clinical stratification remains at an early stage. Thus, targeting endocrine-metabolic crosstalk may support mechanism-based phenotyping and subtype-informed precision therapy for OA, provided that candidate biomarkers and interventions are validated in prospective clinical studies.

## 1. Introduction

Osteoarthritis (OA) is a chronic degenerative joint disorder characterized primarily by progressive articular cartilage degeneration, often accompanied by synovial inflammation, osteophyte formation and subchondral bone sclerosis (1). Its major clinical manifestations include chronic joint pain, morning stiffness and progressive decline in joint function, all of which markedly impair the quality of life of patients (2). With the aging of the population, the rising prevalence of obesity and the increasing incidence of joint injury, the global burden of OA continues to increase. In 2019, the number of individuals affected by OA was >500 million worldwide (3), and this figure is expected to increase further in the coming decades (4,5). OA not only causes substantial functional disability, but also imposes a considerable socioeconomic burden (6). Traditionally, OA has been regarded as a disease driven mainly by mechanical wear and tear (7,8). However, accumulating evidence currently indicates that OA is a whole-joint disorder involving multiple tissues, including cartilage, synovium and subchondral bone, and is closely associated with metabolic dysregulation (9,10).

**Figure 1:**
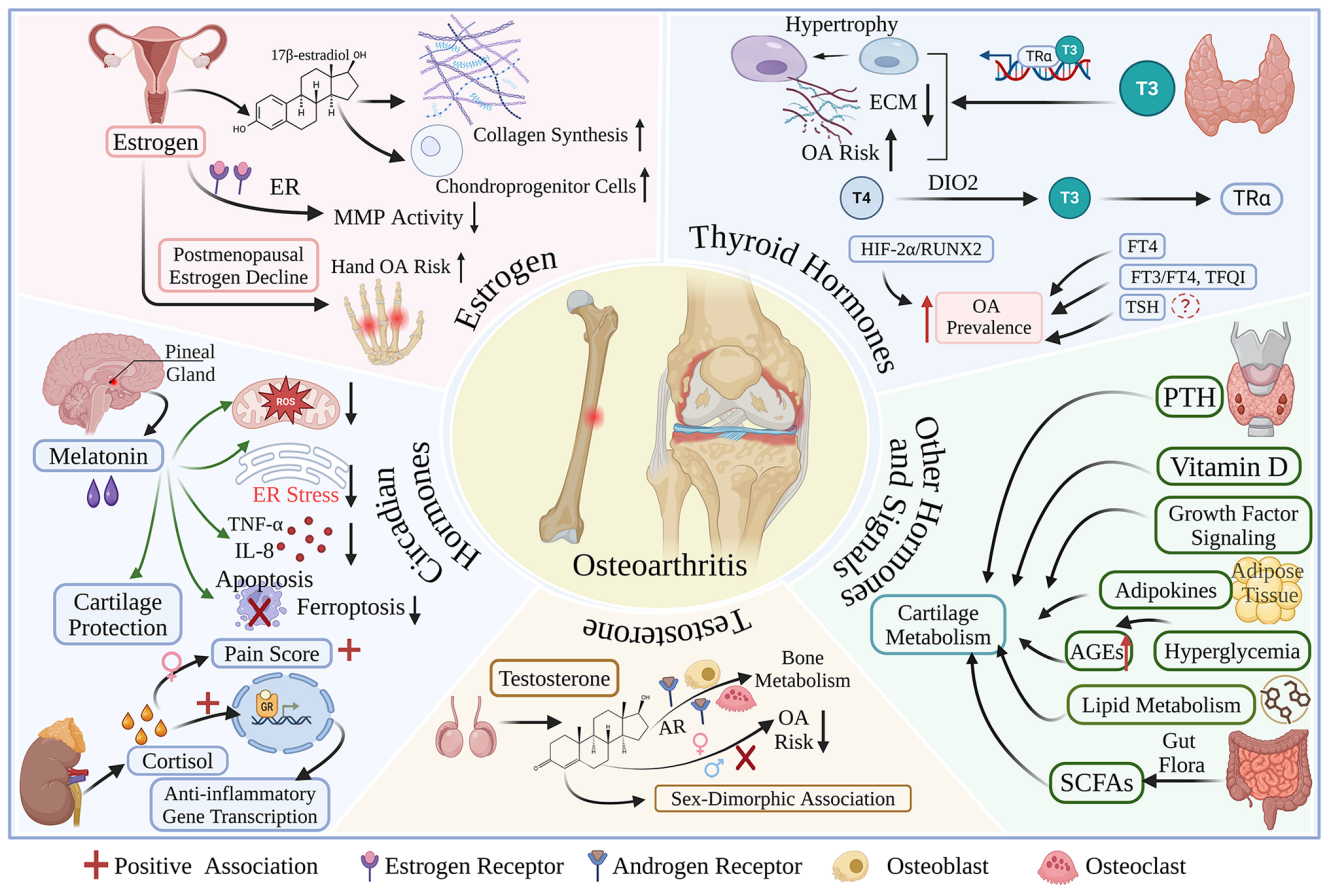
Systemic endocrine-metabolic network driving OA. Sex hormones exert sex-specific effects. Physiological estrogen acting through ERα/β supports chondrogenesis and type II collagen synthesis and limits MMP-driven matrix degradation, whereas the postmenopausal decline in estrogen is associated with a higher risk of developing hand OA in women. Testosterone signaling through the AR modulates bone metabolism and may influence OA risk as well as pain and function in a sex-dependent manner. Thyroid hormone signaling links systemic status to local joint regulation: DIO2-mediated conversion of T4 to T3, followed by TRα activation, promotes chondrocyte hypertrophic differentiation and extracellular matrix breakdown; genetic and epidemiological evidence indicates that higher FT4, together with derived indices such as the FT3-to-FT4 ratio and TFQI, is associated with OA prevalence. Circadian hormones are also integrated, highlighting melatonin-mediated antioxidant and anti-inflammatory actions and protection against cell death pathways including apoptosis and ferroptosis, with potential analgesic benefits, whereas HPA-axis cortisol shows heterogeneous, rhythm-dependent associations with pain and inflammation. Additional endocrine inputs, including parathyroid hormone, vitamin D and growth-factor signaling, converge on cartilage metabolism and subchondral bone remodeling. Metabolic OA pathways include obesity-related adipokines, hyperglycemia and advanced glycation end products with insulin resistance, lipid dysmetabolism and lipotoxicity and the gut-joint axis. OA, osteoarthritis; ERα/β, estrogen receptor α/β; AR, androgen receptor; MMP, matrix metalloproteinase; ECM, extracellular matrix; HIF-2α, hypoxia-inducible factor-2α; RUNX2, Runt-related transcription factor 2; T4, thyroxine (tetraiodothyronine); T3, triiodothyronine; TRα, thyroid hormone receptor alpha; DIO2, type 2 deiodinase; FT4, free thyroxine; FT3, free triiodothyronine; TSH, thyroid-stimulating hormone; TFQI, thyroid feedback quantile-based index; ROS, reactive oxygen species; ER stress, endoplasmic reticulum stress; TNF-α, tumor necrosis factor-α; IL-8, interleukin-8; HPA axis, hypothalamic-pituitary-adrenal axis; GR, glucocorticoid receptor; PTH, parathyroid hormone; AGEs, advanced glycation end products; SCFAs, short-chain fatty acids. The figure was created in BioRender (https://BioRender.com/cyuj626).

Previous research on OA has focused mainly on local pathological processes within the joint, such as cartilage matrix degradation (11), the crosstalk between cartilage and subchondral bone (12) and local inflammatory responses (13). Nevertheless, increasing evidence suggests that the initiation and progression of OA are not driven solely by local factors, but rather reflect a complex process involving disrupted homeostasis across multiple systems, including metabolic (14), immune (15), neural (16), skeletal and intestinal systems (17). In this context, the interplay between systemic metabolic status and the joint microenvironment has attracted increasing attention (18). A large body of experimental and clinical evidence indicates that endocrine hormones and metabolic products can influence joint tissue homeostasis through the circulatory system and regulate key pathological processes, including chondrocyte metabolism, synovial inflammatory responses and subchondral bone remodeling, thereby contributing to the onset and progression of OA (19-25). Based on these observations, the present review adopted the 'endocrine-skeletal axis' as a conceptual framework and highlights the bidirectional regulatory association between the endocrine system and the osteoarticular system. By integrating hormonal signaling, metabolic status and inflammatory responses, this axis mediates the dynamic interplay between systemic metabolism and local joint homeostasis, providing a critical perspective for understanding the systemic pathogenesis of OA. A schematic illustration of the systemic endocrine-metabolic network driving OA is presented in Fig. 1.

The present review aimed to move beyond the conventional single hormone-centered research paradigm, and to systematically summarize the key regulatory roles of hormonal, metabolic and inflammatory networks involved in the pathogenesis and progression of OA from an integrated perspective. Particular emphasis is placed on the interactions between endocrine signaling and metabolic pathways, as well as their influence on major pathological phenotypes, such as cartilage degradation, dysregulated bone remodeling and synovial inflammation. In addition, the present review outlines recent advances in relevant preclinical and clinical studies, and discusses potential therapeutic strategies targeting the endocrine-metabolic interaction network, with the goal of providing a theoretical basis for the precision prevention and treatment of OA.

## 2. Hormonal dysregulation in OA: Epidemiological evidence and pathophysiology

### Sexual dimorphism of sex hormones Estrogen

Evidence suggests that estrogen plays a crucial regulatory role in maintaining the homeostasis of articular cartilage and subchondral bone, and its deficiency can significantly increase the risk of developing OA in postmenopausal women, while accelerating disease progression (26-28). Within the physiological concentration range, 17β-estradiol promotes the chondrogenic potential of cartilage progenitor cells and chondrocytes and enhances the synthesis of key cartilage matrix components, including type II collagen, thereby preserving the normal phenotype of cartilage tissue (29). In addition, estrogen can inhibit matrix degradation mediated by matrix metalloproteinases (MMPs) through estrogen receptor-mediated signaling, thereby reducing cartilage matrix loss and preserving cartilage structural integrity (30).

The expression of estrogen receptor (ER)α and β has been detected in human articular chondrocytes. Although the overall expression levels of these receptors do not differ markedly between normal cartilage and cartilage affected by OA, receptor expression patterns and hormonal sensitivity may exhibit sex-related differences (31,32). Studies have indicated that the decline in estrogen levels after menopause is associated with an increased risk and greater severity of knee and hand OA (33). At the clinical level, previous studies have demonstrated that estrogen replacement therapy may, to a certain extent, alleviate structural degeneration and improve joint symptoms in postmenopausal OA (34-36). Moreover, estrogen levels are negatively associated with the volume of knee effusion-synovitis, and this association appears to be more evident in women than in men, suggesting that estrogen may contribute to sex-related differences in the pathogenesis of OA (37,38).

### Testosterone

Testosterone is a critical sex hormone for maintaining bone metabolic homeostasis. It regulates the functions of osteoblasts and osteoclasts through androgen receptor signaling and thereby participates in the dynamic balance between bone formation and bone resorption (39,40). At present, the association between testosterone and OA remains somewhat controversial. This inconsistency may partly reflect differences in the testosterone indicators examined, including total, free and bioavailable testosterone, as well as age-related hormonal status, sex-specific responses, the site of OA and study design. Genetic analyses suggest that bioavailable testosterone levels may have a causal association with the risk of developing OA (41), whereas epidemiological research has reported that low testosterone levels are associated with an increased risk of developing OA (42). These findings suggest that differences in study populations and OA subtypes may influence the observed associations. Therefore, testosterone-related associations should be interpreted in a site- and population-specific manner.

As regards sex differences, some studies have found that endogenous testosterone levels are negatively associated with the risk of developing knee OA in middle-aged and older women, whereas no similar association has been observed in men (43,44). In addition, testosterone levels may also be associated with the severity of pain and functional impairment in patients with OA (45). For example, in patients undergoing total knee arthroplasty, higher total testosterone levels have been shown to be associated with improved post-operative pain relief and functional recovery (46). Hormonal balances may also affect the risk of developing OA. Evidence suggests that the testosterone-to-estradiol ratio is negatively associated with risk of developing OA, and this association appears to be more pronounced in women (47). Furthermore, chondrocytes exhibit sex-specific responses to androgens and estrogens, indicating that sex hormone signaling may contribute to sex-specific pathological processes in OA (48).

At the level of clinical intervention, the safety of testosterone replacement therapy has been preliminarily evaluated in certain orthopedic populations. For example, as previously demonstrated, in patients undergoing reverse shoulder arthroplasty, short-term preoperative testosterone replacement therapy did not significantly increase the risk of revision, infection, or fracture (49). Existing research also suggests that testosterone replacement therapy may influence a range of OA-related orthopedic outcomes, including the incidence of joint replacement, post-operative recovery and bone healing; however, its overall benefits and risks warrant further investigation (50).

### Thyroid hormones and bone-cartilage crosstalk

Thyroid hormones, with triiodothyronine (T3) as the principal active form, participate in chondrocyte differentiation, endochondral ossification and adult bone remodeling through thyroid hormone receptor α and the deiodinase system. Dysfunction of this axis is closely associated with the onset and progression of OA (51). Mechanistic analyses have demonstrated that T3 can induce chondrocyte hypertrophic differentiation and promote the expression of ossification-related genes, thereby accelerating cartilage matrix degradation and driving OA progression (52). Genetic studies have further suggested a possible causal association between thyroid dysfunction and the risk of developing OA. For example, genetic susceptibility to thyrotoxicosis has been associated with an increased risk of developing knee OA, whereas no similar association has been observed for hip OA (53). In population-based studies, elevated free thyroxine (FT4) levels have been associated with an increased prevalence, greater disease severity and a higher risk of progression of knee OA. This association appears to be more pronounced in individuals with obesity or those exposed to high mechanical loading, whereas the association between thyroid-stimulating hormone and OA remains inconclusive (54). In addition, several indicators reflecting thyroid hormone sensitivity, including the free triiodothyronine (FT3)/FT4 ratio and the thyroid feedback quantile-based index (TFQI), have also been reported to be associated with OA prevalence, with the TFQI exhibiting stronger predictive performance in certain populations (55). Notably, a local thyroid hormone regulatory network has been identified in the synovial tissue of patients with OA. The inflammatory cytokine, tumor necrosis factor-α (TNF-α) can regulate the expression of deiodinases and thyroid hormone receptors, suggesting potential crosstalk between inflammation and thyroid hormone signaling (56).

The deiodinase family, including types I, II and III deiodinase (DIO1, DIO2 and DIO3, respectively), constitutes a key enzymatic system that regulates the local activation and inactivation of thyroid hormones and plays an important role in OA pathogenesis (57). Among these, DIO2 has been regarded as a potential susceptibility gene for OA, and its polymorphisms have been associated with disease risk (58). It has been suggested that, in certain genetic backgrounds, upregulated DIO2 expression may promote cartilage matrix degradation and cartilage mineralization through pathways, such as hypoxia-inducible factor-2α (HIF-2α) and runt-related transcription factor 2, thereby accelerating the progression of OA, whereas the inhibition of related deiodinase activity may help preserve cartilage homeostasis (59). In addition, the D2 enzyme encoded by DIO2 is responsible for converting thyroxine into T3, and its dysfunction may disrupt local thyroid hormone homeostasis and increase the risk of developing OA (60). Clinical studies further suggest that, in individuals at a high risk of developing knee OA, levothyroxine treatment may be associated with a reduced quadriceps muscle mass, and this decline in muscle mass may further alter joint loading and promote the development of OA (61).

### Circadian hormones

Melatonin is a key hormone involved in maintaining circadian homeostasis, and abnormal melatonin secretion has been implicated in the onset and progression of OA (62,63). A growing body of evidence indicates that melatonin exerts multiple protective effects against OA, including antioxidant, anti-inflammatory and metabolic regulatory actions. Mechanistic studies suggest that melatonin can inhibit chondrocyte apoptosis and ferroptosis by regulating mitochondrial function and endoplasmic reticulum homeostasis, while also reducing the release of pro-inflammatory cytokines, such as TNF-α and interleukin (IL)-8 and suppressing the activity of MMPs, thereby preserving cartilage matrix homeostasis (64-68). At the molecular level, melatonin can regulate oxidative stress and inflammatory responses through melatonin receptor 1 and downstream intracellular signaling pathways, thereby attenuating OA-related cartilage injury (69-71). In addition, some clinical studies suggest that melatonin may have analgesic potential, as its use has been found to be associated with reduced subsequent analgesic requirements and a lower risk of joint replacement (72). Current basic and animal studies generally support the potential protective role of melatonin in OA (73-76), and further suggest that it may serve as a candidate agent for OA prevention or treatment (77). However, the available evidence is still derived mainly from experimental studies, and large-scale clinical investigations remain limited. Its precise efficacy, optimal dosage and target populations therefore require further clarification (78).

In addition to melatonin, hormones associated with the hypothalamic-pituitary-adrenal axis may also participate in the pathological process of OA. It has been demonstrated that the association between cortisol and OA is markedly heterogeneous and may be influenced by factors, such as measurement methods, sex and population characteristics (79). In population-based studies, pain severity in women with OA has been reported to be positively associated with cortisol levels (80). By contrast, in patients with knee OA, serum cortisol levels have also been reported to be negatively associated with knee pain scores, and to be associated with the levels of multiple inflammatory mediators (81). Moreover, some studies have found that patients with knee OA exhibit a blunted cortisol awakening response and reduced morning cortisol levels after waking. This circadian alteration is associated with greater pain interference and a lower pain threshold, and may also exhibit sex-related differences (82). At the molecular level, cortisol may exert anti-inflammatory effects by regulating glucocorticoid receptor-related transcriptional activity, thereby contributing to the modulation of inflammatory responses in OA (83). As cortisol secretion displays pronounced circadian rhythmicity, it may exert distinct biological effects across different concentrations and time phases. For example, high cortisol levels may promote inflammatory responses, whereas moderate levels may help maintain the balance between stress adaptation and inflammation (63).

### Other key hormones

Parathyroid hormone (PTH) plays a central role in the regulation of bone metabolism and its association with OA appears to be complex. Genetic studies suggest that elevated serum levels of PTH may have a potential causal association with a reduced risk of developing both hip and knee OA, with the association appearing to be more pronounced in knee OA (84,85). Experimental studies have also shown that PTH (1-34) can attenuate cartilage degeneration and improve subchondral bone microarchitecture in animal models of OA, thereby helping to maintain joint tissue homeostasis (86,87). Mechanistic evidence further suggests that PTH may exert protective effects by regulating chondrocyte proliferation, matrix synthesis and inflammatory responses (88). However, some studies have proposed that PTH may also promote the progression of OA by altering local bone metabolism and the joint microenvironment (89), indicating that its effects may be context-dependent and closely related to the mode of administration and disease stage.

Vitamin D also plays an important role in the regulation of bone and cartilage metabolism, although its value in the prevention and treatment of OA remains controversial. Epidemiological studies have shown that vitamin D deficiency is common in patients with knee OA and is associated with multisite joint pain and greater disease severity (90). At the molecular level, vitamin D may contribute to the maintenance of cartilage homeostasis by regulating chondrocyte autophagy and metabolic pathways (91). However, multiple clinical studies have demonstrated that vitamin D supplementation has limited effects on OA-related pain and functional improvement, and has not demonstrated clear protective benefits against structural changes, such as cartilage volume loss or joint space narrowing (92,93). Therefore, although vitamin D deficiency may be associated with the onset of OA, consistent evidence is still lacking as to whether vitamin D supplementation can delay disease progression (94).

In addition to the hormones described above, several growth factor-related signaling pathways may also contribute to the onset and progression of OA. For example, growth hormone and its downstream mediator insulin-like growth factor-1 are involved in the maintenance of joint homeostasis through the regulation of bone metabolism and chondrocyte function, and abnormal levels of these factors may affect bone microarchitecture and increase the risk of developing OA (95). In addition, growth hormone-releasing hormone has been reported to promote chondrocyte proliferation and enhance extracellular matrix (ECM) synthesis, thereby exerting protective effects against cartilage injury (96). The transforming growth factor-β (TGF-β) family plays an essential role in cartilage development and tissue repair, and its biological effects may be concentration-dependent. Low levels appear to support cartilage homeostasis, whereas aberrant activation may aggravate joint structural damage (97,98). Vascular endothelial growth factor (VEGF) is closely associated with OA-related angiogenesis and pain regulation, and its abnormal expression may promote cartilage degeneration and contribute to OA pain development (99).

## 3. Metabolic syndrome and metabolic OA phenotype

### Concept and clinical phenotype of metabolic OA

Metabolic OA is increasingly recognized as a key clinical subtype of OA, and its onset and progression are closely associated with metabolic syndrome and related metabolic disturbances. This subtype is typically driven by the combined effects of obesity, insulin resistance, dyslipidemia and chronic low-grade inflammation. Through the synergistic interaction between systemic metabolic abnormalities and alterations in the local joint microenvironment, these factors ultimately lead to structural joint degeneration and functional impairment (100).

From a clinical perspective, metabolic OA is most commonly observed in individuals aged 45 to 65 years, frequently affects the knee and hand joints, and is often characterized by more pronounced inflammatory responses and pain symptoms. Patients with this subtype commonly present with metabolic comorbidities, such as obesity, diabetes and cardiovascular disease, suggesting that the development of OA is not determined solely by local joint factors, but is also closely linked to systemic metabolic status (101). Epidemiological research has demonstrated that, in middle-aged women, the severity of metabolic syndrome is significantly associated with the structural progression of knee OA over subsequent years, including osteophyte formation, bone marrow lesions and cartilage defects. Among these factors, indicators, such as waist circumference, blood glucose levels and high-density lipoprotein (HDL) cholesterol may serve as critical predictors (102).

In recent years, it has become increasingly evident that metabolic abnormalities can disrupt joint tissue homeostasis through multiple mechanisms, including systemic inflammation, immune dysregulation and altered metabolic signaling pathways. Examples include gut microbiota imbalances, changes in immune cell polarization and the dysregulation of lipid metabolism-related signaling pathways (102-105). These mechanisms provide a key theoretical basis for understanding the development and progression of metabolic OA, and may also provide potential directions for future biomarker discovery and the development of targeted intervention strategies.

### Obesity and adipokines: From mechanical loading to metainflammation

Obesity is one of the most critical modifiable risk factors for OA. Its impact on OA arises not only from the increased mechanical loading associated with excess body weight, but also from the metabolic inflammation mediated by a wide range of adipokines secreted by adipose tissue (106,107). Adipose tissue dysfunction can lead to dysregulated adipokine secretion and trigger chronic low-grade inflammation, thereby affecting cartilage metabolism, synovial inflammation and subchondral bone remodeling. This process is considered a major pathological basis of obesity-related OA (107,108). Among these adipokines, leptin is one of the most extensively studied. Its levels are significantly elevated in patients with obesity and OA, and it can promote the expression of pro-inflammatory mediators and MMPs through the activation of signaling pathways, such as the JAK/signal transducer and activator of transcription (STAT) pathway and nuclear factor κB (NF-κB) signaling, while simultaneously suppressing cartilage matrix synthesis. In this manner, leptin accelerates cartilage degradation and contributes to synovitis and abnormal bone remodeling (21,23). Epidemiological research has further demonstrated that serum leptin levels are positively associated with the risk of developing hand and knee OA, and may partly explain the link between obesity and OA. This effect appears to be more evident in women and in individuals with knee OA (109).

In addition to leptin, several other adipokines are involved in the pathological process of obesity-related OA. For example, adiponectin may exert bidirectional and context-dependent effects in OA. On the one hand, it participates in metabolic regulation through pathways, such as AMP-activated protein kinase (AMPK). On the other hand, it may promote inflammatory responses and influence chondrocyte apoptosis and autophagy (110,111). Adipokines, such as visfatin and resistin are upregulated in obesity and can aggravate joint tissue injury by promoting the expression of inflammatory mediators and matrix-degrading enzymes. Their levels have also been associated with OA severity (107,112). Notably, adipokines are involved not only in structural joint degeneration, but also in the regulation of OA pain through peripheral inflammation and mechanisms related to neural sensitization (108). Taken together, adipose tissue in OA is not merely a source of excessive mechanical loading, but also an important endocrine organ, and the adipokines it secretes play a central role in linking systemic metabolic abnormalities with local joint inflammation.

### Glucotoxicity and insulin resistance: Metabolic-inflammatory reprogramming drives the progression of OA

Glucotoxicity and insulin resistance are increasingly recognized as key pathological links underlying the comorbidity of type 2 diabetes mellitus (T2DM) and OA. Through the interplay between metabolic dysregulation and inflammatory responses, these processes jointly promote joint degeneration. Chronic hyperglycemia can disrupt the homeostasis of the joint microenvironment through multiple mechanisms. For example, high glucose levels can enhance glycolysis in synovial macrophages and promote lactate accumulation, thereby inducing CD11b lactylation and impairing macrophage efferocytosis, which in turn aggravates synovial inflammation. In this process, the acetyltransferase CREB-binding protein may serve as a critical mediator, and targeted CREB-binding protein knockdown has been shown to partially delay the progression of hyperglycemia-associated OA (113). At the same time, hyperglycemia can promote the accumulation of advanced glycation end products in fibroblast-like synoviocytes (FLSs) through the HIF-1α/glucose transporter 1 (GLUT1) pathway and induce endoplasmic reticulum stress, further increasing the expression of inflammatory mediators, such as TNF-α and IL-6, as well as matrix-degrading enzymes including MMPs and a disintegrin and metalloproteinase with thrombospondin motifs (ADAMTS). These changes suppress cartilage matrix synthesis and accelerate matrix degradation (114). In addition, high-glucose conditions can induce oxidative stress and exacerbate joint tissue injury, further supporting glucotoxicity as a key mechanistic link between metabolic abnormalities and joint inflammation (19).

Epidemiological studies also support a critical role for insulin resistance in the development of OA. Multiple population-based studies have shown that insulin resistance-related indicators, including the triglyceride-glucose index, its derived indices and the metabolic score for insulin resistance, are all significantly associated with the risk of developing OA. These findings suggest that insulin resistance may serve as a key metabolic marker linking metabolic syndrome to the onset of OA, and may provide a potential basis for early screening and risk assessment (115-118). At the molecular level, abnormal insulin signaling can amplify inflammatory responses and suppress autophagy through pathways, such as phosphoinositide 3-kinase (PI3K)/protein kinase B (Akt)/mechanistic target of rapamycin (mTOR)/NF-κB, thereby promoting the secretion of inflammatory mediators and chemokines by FLSs and upregulating cartilage-degrading molecules such as MMP-9 and MMP-13, which ultimately aggravates synovial inflammation and cartilage damage (119). Notably, OA and T2DM share a common metabolic background characterized by nutrient excess, chronic inflammation, hyperglycemia and mitochondrial dysfunction. Among the key regulatory nodes involved, AMPK has been highlighted as a central integrator linking energy metabolism, inflammatory signaling, and cellular stress responses across these pathological processes (120).

At the clinical level, T2DM and poor glycemic control have been reported to be significantly associated with an increased risk of symptomatic knee OA, independently of traditional risk factors such as age and body mass index, suggesting that glycemic management may represent a key strategy for preventing adverse OA-related outcomes (121). In addition, several widely used glucose-lowering agents, such as metformin and glucagon-like peptide-1 receptor agonists, are considered to exert potential protective effects against OA by improving metabolic inflammation, regulating energy metabolism and suppressing cartilage degradation. However, their precise therapeutic value still requires further confirmation in clinical studies (122).

### Dysregulated lipid metabolism and lipotoxic injury

The association between dyslipidemia and OA remains somewhat heterogeneous. Epidemiological research suggests that dyslipidemia is associated with an increased risk of developing OA, although the findings are not fully consistent across different study designs (123). For example, some studies have reported that dyslipidemia is associated with a higher risk of developing hand OA, with the association for elevated levels of triglycerides appearing to be relatively consistent, whereas the evidence linking low-density lipoprotein (LDL) and HDL levels to hand OA remains inconclusive (124). At the same time, several studies have not identified a clear association between dyslipidemia and knee OA (125). In addition, lipid-related indices may also be associated with OA pain phenotypes. For instance, HDL cholesterol may exert a protective effect, whereas elevated triglycerides may increase risk (126). Overall, dyslipidemia may contribute to the association between obesity and OA by promoting systemic inflammatory responses, but its causal role and clinical significance still require further clarification (123,127).

To more directly synthesize the inconsistent epidemiological findings, current evidence suggests that elevated levels of triglycerides exhibit relatively consistent positive associations with OA, particularly in studies on hand OA and OA-related pain phenotypes (124,126). By contrast, although HDL cholesterol may be associated with certain OA-related pain phenotypes, its association with structural OA remains inconsistent (124,126), whereas LDL cholesterol and total cholesterol have not exhibited a uniform association with OA across studies (123,124). The association between dyslipidemia and OA also appears to be joint-site specific, being more evident for hand OA than for knee OA, for which some studies have reported no clear association (124,125). This heterogeneity may partly reflect differences in the joint site examined, the lipid parameters assessed, outcome definitions and study design, including cross-sectional, case-control and cohort approaches (123-126).

Beyond circulating lipid levels, the local dysregulation of lipid metabolism within the joint is increasingly regarded as a key mechanism underlying the onset and progression of OA. Disrupted cholesterol homeostasis in chondrocytes can impair ECM homeostasis through several mechanisms. For example, defective cholesterol efflux or abnormal cholesterol metabolic pathways can activate signaling cascades, such as Ras/Raf/MEK/ERK, thereby promoting cartilage matrix degradation (128,129). In addition, abnormal fatty acid metabolism is also involved in the pathogenesis of OA. In obesity-related OA, enhanced fatty acid oxidation (FAO) in chondrocytes can aggravate abnormalities in cartilage matrix metabolism through metabolic reprogramming and epigenetic regulation (130). Different types of fatty acids exert distinct effects on OA. Among these, n-3 polyunsaturated fatty acids, such as eicosapentaenoic acid and docosahexaenoic acid, may exert protective effects through pathways including peroxisome proliferator-activated receptor γ and NF-κB, whereas n-6 fatty acids may promote inflammatory responses and accelerate cartilage degradation (131). Moreover, lipid peroxidation is considered a critical mechanism of cartilage injury. For example, lipid peroxidation mediated by acyl-CoA synthetase long-chain family member 4 can induce ferroptosis and amplify joint inflammatory responses (132). Taken together, abnormal lipid metabolism not only affects systemic inflammatory status, but also disrupts cartilage homeostasis through local metabolic reprogramming.

Oxidized LDL (oxLDL) is considered a key molecular link between lipid metabolic abnormalities and OA. Upon binding to lectin-like oxidized low-density lipoprotein receptor-1, oxLDL can induce oxidative stress and inflammatory responses, thereby promoting cartilage degeneration and osteophyte formation (133,134). In chondrocytes, oxLDL can suppress transcription factor EB activity through the activation of the ERK1/2 and mTOR pathways, leading to impaired autophagy and lysosomal dysfunction and ultimately inducing cell death (135). In synovial tissue, oxLDL can activate macrophages and fibroblasts, further aggravating synovial inflammation (136,137). Based on these mechanisms, the modulation of lipid metabolism or the inhibition of related signaling pathways has been considered to hold therapeutic potential. For example, statins or agents targeting the mTOR pathway have been reported in some studies to possibly attenuate the progression of OA, although their clinical efficacy warrants further validation (135).

### The Gut-Joint axis: An upstream regulator of metabolic and endocrine signaling

Gut microbiota dysbiosis is increasingly regarded as a crucial initiating factor that drives the dysfunction of the gut-joint axis and contributes to the onset and progression of OA. Studies have shown that the composition of the gut microbiota is significantly altered in patients with OA, as reflected by an increased abundance of potentially pathogenic bacteria, such as *Actinomycetaceae* and *Bilophila*, together with a reduction in the numbers of beneficial microbes, including *Roseburia* and *Bifidobacterium*. These changes are accompanied by abnormalities in amino acid, carbohydrate and lipid-related metabolic pathways (138-140). Alterations in microbial composition not only impair intestinal metabolic function, but can also promote systemic inflammation by disrupting the intestinal barrier. For example, microbial dysbiosis can increase zonulin levels and downregulate the expression of tight junction proteins, such as zonula occludens-1 and occludin, thereby increasing intestinal permeability. At the same time, it can induce the release of inflammatory mediators including TNF-α and IFN-γ, and weaken the structural integrity of the mucus barrier (138,139,141). Under these conditions, bacterial metabolites, such as lipopolysaccharide (LPS) and other inflammatory mediators can enter the circulation and trigger low-grade inflammatory responses, which are considered an important mechanism linking gut microbiota abnormalities to OA-related inflammation.

Short-chain fatty acids (SCFAs) produced by the gut microbiota are considered key molecular mediators regulating the gut-joint axis. Among these, acetate, propionate and butyrate account for the vast majority of total intestinal SCFAs, and can regulate immune responses and intestinal barrier function through mechanisms, such as the activation of G protein-coupled receptors (GPRs), including GPR41, GPR43 and GPR109A, as well as the inhibition of histone deacetylases. Through these actions, SCFAs help maintain the Treg/Th17 balance and regulate macrophage polarization (142-146). Among all SCFAs, butyrate appears to exert particularly prominent anti-inflammatory effects. It can inhibit the NF-κB and mitogen-activated protein kinase (MAPK) signaling pathways through GPR43, reduce the expression of inflammatory and matrix-degrading molecules, such as IL-1β, TNF-α and MMP13, and improve chondrocyte autophagy, as well as type II collagen expression (147-149). Clinical research further suggests that butyrate supplementation may, to a certain extent, improve pain and functional scores in patients with knee OA (150). In addition, interventions, such as high-fiber diets, probiotic or prebiotic supplementation and fecal microbiota transplantation may improve the intestinal metabolic environment by increasing SCFA levels, thereby providing potential avenues for the prevention and treatment of OA (144). A summary of key endocrine and metabolic mediators involved in the pathogenesis of OA is presented in Table I.

## 4. Cell fate pathways driven by immunometabolic stress

### Immunometabolic low-grade inflammation

**Figure 2:**
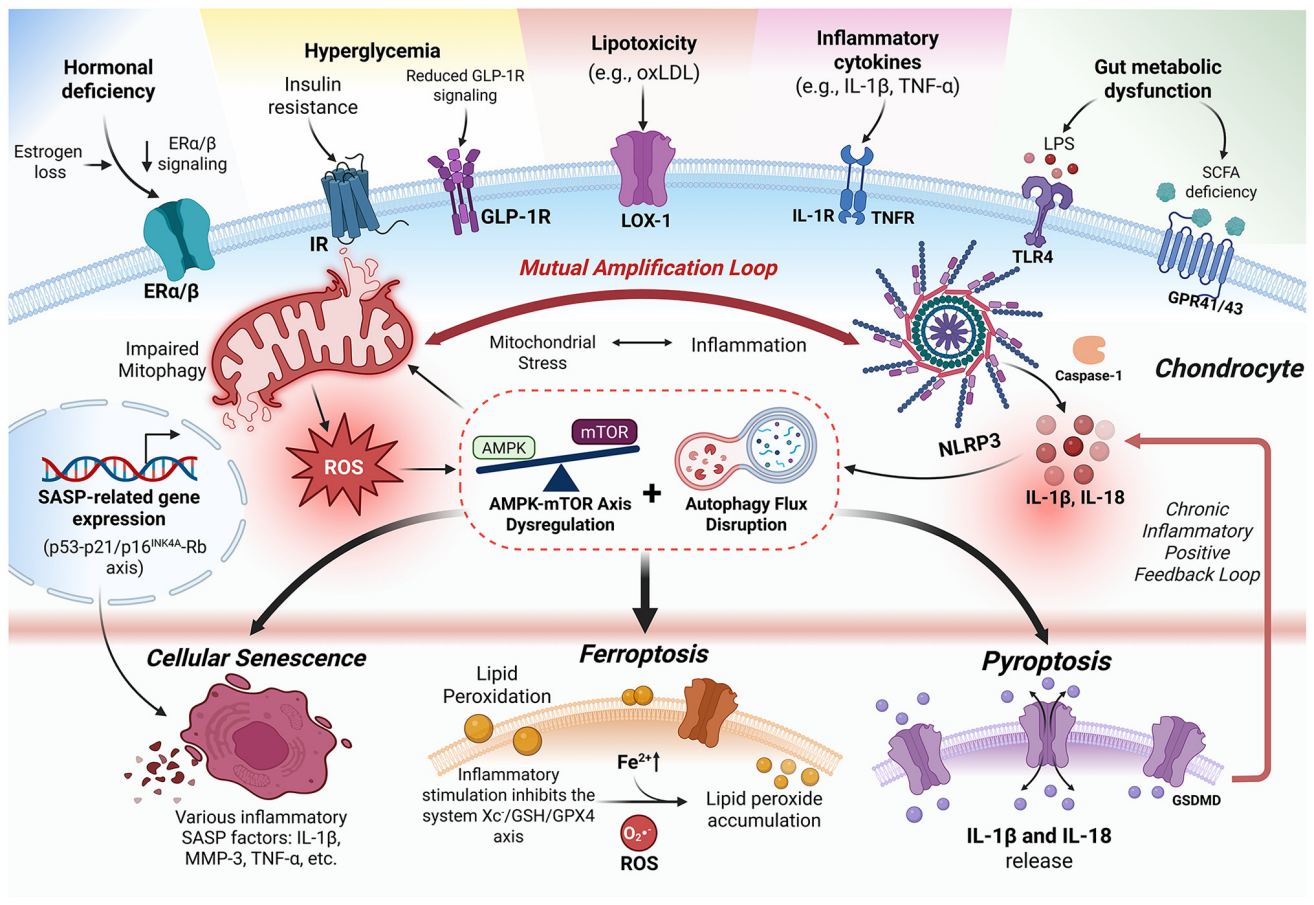
Immunometabolic stress links endocrine-metabolic imbalance to chondrocyte degeneration in OA. The image illustrates the convergence of hormonal and metabolic stressors within chondrocytes, driving inflammatory amplification and degenerative cell-fate transitions in OA. Upstream disturbances include estrogen loss, impaired insulin signaling, dyslipidemia, reduced GLP-1R signaling, gut microbiota dysbiosis, inflammatory stimulation and mechanical stress. The schematic diagram is organized from top to bottom into three conceptual layers: Signal perception, stress integration and cellular outcomes. Extracellular upstream stimuli are sensed by specific receptors on the cell membrane. These signals converge intracellularly on two central, interconnected stress hubs, mitochondrial dysfunction and NLRP3 inflammasome activation, which engage in a positive feedback loop. This leads to the dysregulation of the central AMPK-mTOR axis and disruption of autophagy flux. Ultimately, these integrated stresses drive three major cell fate outcomes: Pyroptosis, ferroptosis and cellular senescence. Ferroptosis is regulated in part by the system Xc^−^/GSH/GPX4 axis and ROS/lipid peroxide accumulation, whereas pyroptosis involves inflammasome activation, caspase-1 and the release of IL-1β/IL-18. All three outcomes contribute to amplifying the inflammatory response, thereby establishing a chronic inflammatory feedback loop that exacerbates the initial pathological state. OA, osteoarthritis; AMPK, AMP-activated protein kinase; ERα/β, estrogen receptor α/β; GLP-1R, glucagon-like peptide-1 receptor; GPX4, glutathione peroxidase 4; GPR, G protein-coupled receptor; GSDMD, gasdermin D; IR, insulin receptor; LOX-1, lectin-like oxidized low-density lipoprotein receptor-1; MMP, matrix metalloproteinase; mTOR, mechanistic target of rapamycin; NLRP3, NLR family pyrin domain-containing 3; SASP, senescence-associated secretory phenotype; TLR4, Toll-like receptor 4; TNFR, tumor necrosis factor receptor. The figure was created in BioRender (https://BioRender.com/x282hxr).

In OA, metabolic abnormalities can function as upstream drivers of low-grade inflammation. A central feature of this process is the imbalance between ECM synthesis and degradation in cartilage (152,153). Immunometabolic reprogramming further amplifies this imbalance. For example, pyruvate kinase M2-mediated glycolytic activation and microRNA-576-5p deficiency can induce chondrocyte stress and promote the release of damage-associated molecular patterns (DAMPs), including adenosine triphosphate (ATP), high mobility group box 1 and S100A8/A9 (152-155). These DAMPs activate pattern recognition receptors, such as Toll-like receptor 4 and NLR family pyrin domain-containing 3 (NLRP3), leading to the activation of NF-κB, MAPK and inflammasome signaling. This response increases MMP expression, chondrocyte apoptosis and the release of IL-1β, thereby sustaining low-grade inflammation within the joint (152-156). Thus, immunometabolic reprogramming establishes an inflammation-related positive feedback loop that contributes to the progression of chronic OA. Enhanced glycolysis and reactive oxygen species (ROS) accumulation are central metabolic nodes involved in this process (157). The link between immunometabolic stress and endocrine-metabolic imbalance with chondrocyte degeneration in OA is illustrated in Fig. 2.

Within the OA microenvironment, multiple stimuli, including inflammatory cytokines, hyperglycemia and mechanical injury, can induce metabolic reprogramming in synovial cells and chondrocytes, and establish an inflammation-amplifying network centered on glycolysis. For example, under IL-1β stimulation, exosomes released by inflammatory FLSs can be taken up by macrophages and enhance HIF1A activity, thereby driving the expression of key glycolytic enzymes such as GLUT1 and hexokinase 2 and increasing glycolytic flux in macrophages (158). At the same time, chondrocytes exposed to IL-1β activate aerobic glycolysis through the NF-κB pathway and upregulate lactate dehydrogenase A expression, exhibiting metabolic features reminiscent of the Warburg effect (159). In addition, hyperglycemia can directly promote glycolysis in synovial macrophages and enhance lactate production, thereby further amplifying metabolic dysregulation (113). Taken together, these findings indicate that glycolytic reprogramming is a critical link connecting inflammatory stimulation with immunometabolic imbalance.

Enhanced glycolysis leads to the accumulation of metabolic byproducts, such as lactate and ROS, which further amplify inflammatory responses through multiple mechanisms. For example, lactate can upregulate nicotinamide adenine dinucleotide phosphate oxidase 4 (NOX4) and promote ROS generation through HCAR1/PI3K/Akt signaling, while ROS in turn activates the NLRP3 inflammasome and induces the release of inflammatory mediators (159-162). Moreover, increased glycolysis in macrophages promotes the secretion of IL-1β and TNF-α, which further stimulates FLSs to release more inflammation-related exosomes, thereby forming a sustained inflammatory amplification loop (158). ROS and associated inflammatory signaling can also promote MMP expression and aggravate cartilage damage, leading to the release of additional DAMPs and further reinforcing metabolic abnormalities. This positive feedback network enables inflammation to persist beyond the initial trigger and ultimately drives the progression of OA (159). Therefore, targeting key glycolytic nodes, such as HIF1A and lactate dehydrogenase A, or ROS-related pathways, including NOX4 and NLRP3, may represent promising strategies for intervening in immunometabolic inflammation in OA.

### Energy crisis and disrupted mitochondrial quality control

The inflammatory microenvironment, including IL-1β and TNF-α, together with metabolic stressors, such as mechanical loading, obesity and aging, can synergistically induce disturbances in chondrocyte energy metabolism and are considered key triggers of mitochondrial dysfunction in OA (163-165). These stimuli can disrupt cellular energy regulatory networks and affect the AMPK-mTOR signaling axis, thereby altering the levels of autophagy and mitophagy. As a key sensor of cellular energy homeostasis, AMPK is activated through phosphorylation at the Thr172 site of its α subunit and promotes autophagy initiation by inhibiting mTOR activity, thereby helping to maintain energy balance (166-168). However, under OA-related metabolic stress, this regulatory axis is often impaired. For example, insufficient β-hydroxybutyrate (βOHB) can weaken HCAR2-mediated AMPK activation and suppress PTEN-induced putative kinase 1 (PINK1)/Parkin-dependent mitophagy, thereby impairing the clearance of damaged mitochondria (167). By contrast, excessive enhancement of FAO may disrupt cartilage matrix homeostasis by suppressing AMPK activity and promoting SRY-box transcription factor 9 (SOX9) degradation (130). Taken together, these findings suggest that the AMPK-mTOR axis serves as a key regulatory node linking metabolic stress to mitochondrial quality control.

Impaired autophagy and mitophagy directly disrupt mitochondrial quality control and promote the accumulation of oxidative stress. When mitophagy is insufficient, damaged mitochondria progressively accumulate within cells, manifesting as cristae disruption, loss of membrane potential and reduced ATP production, together with excessive generation of ROS (168). Metabolic regulators, such as α-ketoglutarate (α-KG) and sirtuin (SIRT)4 are also involved in this process, and alterations in their expression can aggravate mitochondrial fragmentation and promote ROS production (169,170). Furthermore, ROS not only damages mitochondrial DNA and respiratory chain complexes, but also reinforces the vicious cycle of mitochondrial dysfunction, ROS accumulation and autophagic imbalance through feedback regulation of the AMPK-mTOR pathway (171).

This energy crisis and oxidative stress ultimately translate into metabolic imbalance of the cartilage ECM. For example, ROS can activate inflammatory signaling and upregulate the expression of MMP13 and ADAMTS5, while suppressing the synthesis of collagen type II alpha 1 chain (Col2a1) and aggrecan, thereby promoting cartilage degeneration (169). In addition, enhanced FAO may further inhibit the expression of ECM synthesis-related genes through epigenetic reprogramming (130). Therefore, modulation of the AMPK-mTOR signaling axis or restoration of mitochondrial metabolic homeostasis, such as by targeting FAO or supplementing metabolites, including βOHB and α-KG, may represent promising strategies for alleviating metabolic abnormalities and cartilage injury in OA (130,167).

### Programmed cell death (PCD)

The progression of OA is closely associated with the aberrant activation of PCD in chondrocytes. Accumulating evidence indicates that multiple forms of PCD are involved in OA, including apoptosis, pyroptosis, ferroptosis, necroptosis, autophagy, cuproptosis and PANoptosis (172-174). Through complex molecular networks, these cell death modalities collectively influence chondrocyte survival and function, and contribute to inflammatory amplification and cartilage matrix destruction. Among these, pyroptosis and ferroptosis have attracted particular attention in recent years due to their close links to inflammatory responses and oxidative stress, as well as their potential therapeutic relevance. To clarify these overlapping pathways, Table II summarizes their major triggers, molecular mediators, OA-related consequences, current evidence status and provides supporting references.

Pyroptosis is a form of PCD accompanied by a robust inflammatory response and can contribute to OA-related cartilage injury through both canonical, namely caspase-1-dependent, and non-canonical, namely caspase-4/5/11-dependent, pathways (175). In the canonical pathway, stimuli such as IL-1β, TNF-α, LPS, ATP, ROS and mechanical stress can activate NF-κB or MAPK signaling, thereby inducing the expression of NLRP3 inflammasome-related components and promoting inflammasome assembly, followed by the recruitment and activation of caspase-1. Activated caspase-1 cleaves gasdermin D (GSDMD), resulting in the formation of membrane pores, while also promoting the maturation and release of IL-1β and IL-18, thereby amplifying inflammatory responses (176). The non-canonical pathway is initiated by the direct activation of caspase-4/5 in humans or caspase-11 in mice by cytosolic LPS, and similarly induces pyroptosis through GSDMD cleavage (177). Both pathways ultimately converge on GSDMD-mediated membrane rupture and inflammatory cytokine release, which further upregulate the expression of matrix-degrading enzymes such as MMP1, MMP3, MMP13 and ADAMTS4/5, thereby suppressing cartilage matrix synthesis and aggravating synovitis and cartilage degeneration (178).

Ferroptosis is a form of PCD driven by iron overload and lipid peroxidation, with the core regulatory axis centered on system Xc^−^/glutathione (GSH)/glutathione peroxidase 4 (GPX4) (179). System Xc^−^ is composed of solute carrier family (SLC)7 member 11 (SLC7A11) and SLC3A2, and maintains GSH synthesis through cystine-glutamate exchange, whereas GSH serves as an essential cofactor for GPX4 to reduce lipid peroxides and limit oxidative injury (180). In the OA microenvironment, inflammatory stimulation or mechanical stress can suppress SLC7A11 expression and reduce GPX4 activity, thereby leading to the gradual accumulation of lipid peroxides (181). At the same time, dysregulated iron metabolism can further amplify this process. For example, transferrin receptor 1-mediated iron uptake, ferritin degradation, and reduced iron efflux can all increase intracellular free Fe^2+^ levels and promote ROS generation through the Fenton reaction (182). Lipid metabolic reprogramming also provides key substrates for ferroptosis. For instance, acyl-CoA synthetase long-chain family member 4 and lysophosphatidylcholine acyltransferase 3 promote the formation of polyunsaturated fatty acid-phosphatidylethanolamine, which can subsequently undergo peroxidation under the catalysis of lipoxygenases, thereby disrupting membrane stability (183). Once activated, ferroptosis not only induces chondrocyte injury, but also promotes MMP13 expression and suppresses type II collagen synthesis, thereby further aggravating cartilage matrix imbalance and driving OA progression (184).

### Cellular senescence and the senescence-associated secretory phenotype (SASP)

Cellular senescence and the SASP it mediates are increasingly recognized as key drivers of OA progression, and their regulation involves multiple interconnected pathways related to cell cycle control, inflammatory signaling and metabolic stress (191,192). In general, the p53-p21/p16^INK4A^-Rb axis is primarily involved in the initiation of senescence, whereas the mTOR pathway mainly regulates SASP at the translational level, and NF-κB contributes to the transcriptional activation of SASP. In addition, signaling pathways, such as IL-6-STAT3, ROS-MAPK and autophagy-GATA4 also participate in shaping the SASP regulatory network (193,194).

The p53-p21/p16^INK4A^-Rb pathway represents a major regulatory axis of OA-related cellular senescence. Among its components, p21 is considered a critical effector molecule in immune inflammation-induced chondrocyte senescence. It has been demonstrated that IL-17 can upregulate the expression of p21 encoded by cyclin-dependent kinase inhibitor 1A, whereas either p21 knockdown or neutralization of IL-17 can attenuate the senescent phenotype of chondrocytes and improve their chondrogenic capacity (195). As with p21, p16^INK4A^ is also a key marker of post-traumatic senescent cells, and its expression significantly increases with the progression of OA and aging (196,197). However, increasing evidence suggests that p16^INK4A^ may be more suitable as a biomarker of cellular senescence rather than a direct pathogenic factor, since its expression does not appear to be a prerequisite for SASP production (197). In addition, senolytic strategies that selectively eliminate senescent cells can reduce the burden associated with p16^INK4A^, and weaken the reciprocal amplification between senescence and inflammation, thereby alleviating joint tissue degeneration (195,196).

mTOR signaling also plays a key role in the regulation of cellular senescence and SASP in OA. It has been demonstrated that the selective inhibition of mTOR complex 1 can activate Akt signaling through negative feedback and enhance autophagic flux, thereby attenuating IL-1β-induced cellular senescence and reducing the secretion of matrix-degrading SASP factors (198). By contrast, lactate accumulation in the OA microenvironment can promote p53 and p21 expression through the arginase 2-mTOR/ribosomal protein S6 kinase β-1/eukaryotic translation initiation factor 4B signaling cascade and induce G1/S arrest in synovial cells, while also enhancing the secretion of multiple inflammatory SASP factors and aggravating synovial inflammation (199). In addition, fibroblast growth factor 21 can inhibit mTOR phosphorylation through SIRT1 and promote transcription factor EB-mediated autophagy activation, thereby reducing the expression of p16^INK4A^ and p21 and alleviating chondrocyte senescence, while also suppressing SASP secretion and promoting cartilage matrix synthesis (200). Taken together, these findings highlight the critical role of the mTOR-autophagy axis in regulating senescence and the SASP network in OA.

## 5. Therapeutic strategies targeting the endocrine-metabolic axis and future directions

### Strategies for modulating hormonal signaling

The efficacy and risks of hormone replacement therapy (HRT) and selective estrogen receptor modulators (SERMs) in OA exhibit substantial heterogeneity, and their effects are influenced by factors such as the affected joint site, timing of intervention, and route of administration. Current evidence suggests that systemic HRT is associated with an increased risk of the onset of knee OA and joint replacement (201). By contrast, combined estrogen-progestin HRT initiated during the early perimenopausal period may reduce the risk of developing hand OA, although this potential benefit appears to be largely confined to the treatment period, and the risk may rise again following discontinuation (202). A previous meta-analysis of animal models indicated that estrogen therapy can upregulate type II collagen expression and reduce the levels of cartilage degradation markers, including C-terminal telopeptide of type II collagen and cartilage oligomeric matrix protein, thereby ameliorating cartilage degeneration (36). SERMs may also exert protective effects by suppressing cartilage turnover (36). However, clinical studies have demonstrated that HRT does not confer clear advantages in improving hand OA-related function, while long-term systemic use may increase the risk of developing cardiovascular disease and hormone-related malignancies, which limits its broader application in the treatment of OA (203,204). Overall, the available clinical and observational evidence for HRT in OA remains inconsistent and appears to be influenced by joint site, timing of initiation, formulation and treatment duration, whereas the evidence supporting SERMs is still derived mainly from preclinical models.

The therapeutic window is considered a critical determinant of hormonal intervention strategies. It has been demonstrated that estrogen therapy initiated during the early postmenopausal stage can significantly improve cartilage structure and attenuate joint degeneration, with effects that are clearly superior to those achieved with late intervention (36). In population-based studies, the efficacy of HRT also appears to be closely related to formulation type and duration of use. For example, oral formulations have exhibited some advantage in reducing the risk of developing hand OA, whereas patients receiving treatment for ≥5 years tend to exhibit an increased risk of developing knee OA (202). These findings suggest that the potential benefits of hormonal therapy in OA depend on precise population stratification and appropriate timing of intervention.

Compared with systemic administration, intra-articular local delivery strategies can increase local drug concentrations, while minimizing systemic exposure and are therefore emerging as an important technological direction in the treatment of OA. In recent years, the application of nanodelivery systems and hydrogel-based materials has markedly improved intra-articular retention and the controlled release of therapeutic agents. For example, lipid nanoparticles can enhance drug accumulation within the joint cavity and improve the stability and cellular uptake efficiency of mRNA-based therapeutics (205). Liposome-anchored hydrogels can achieve sustained release and prolong *in vivo* activity to ~22 days, while exerting synergistic anti-inflammatory and pro-chondrogenic effects without evident organ toxicity (206). In addition, combined injection of platelet-rich plasma and hyaluronic acid has shown more durable pain relief and functional improvement than either treatment alone (207), whereas chitosan combined with low-dose glucocorticoids or local growth hormone injection has also demonstrated some potential for cartilage protection and repair (208,209). Overall, local delivery systems may improve intra-articular retention and reduce systemic exposure. However, the maturity of evidence varies across platforms. Platelet-rich plasma combined with HA has been evaluated in clinical studies, whereas lipid nanoparticles, liposome-anchored hydrogels, and other advanced delivery systems remain largely preclinical or early translational platforms that require further clinical validation (205-209).

### Drug repurposing guided by metabolic pathways

The potential benefits of glucagon-like peptide-1 receptor agonists (GLP-1 RAs) in OA extend beyond weight reduction and may also involve direct joint-protective effects through multiple weight-independent mechanisms. It has been demonstrated that GLP-1R is expressed in both human knee chondrocytes and synovial tissue (210), providing a molecular basis for local actions. In terms of weight-dependent effects, GLP-1 RAs promote substantial weight loss by suppressing appetite and delaying gastric emptying, thereby reducing mechanical stress on weight-bearing joints and attenuating obesity-related systemic inflammation (211). In terms of weight-independent mechanisms, these agents can suppress NF-κB signaling and reduce the release of pro-inflammatory mediators, such as TNF-α and IL-1β, while also promoting the polarization of macrophages from the M1 to the M2 phenotype and downregulating the expression of cartilage-degrading enzymes, such as MMP-3 and MMP-13 (210-212). Clinically, the STEP-9 trial revealed that after 68 weeks of treatment with semaglutide at 2.4 mg weekly, patients with obesity and OA achieved an additional 14.1-point improvement in the Western Ontario and McMaster Universities Osteoarthritis Index (WOMAC) pain score compared with the patients treated with the placebo (213). In addition, a cohort study suggested that the long-term use of GLP-1 RA was associated with a slower knee cartilage loss and a lower risk of joint replacement (214). Clinically, the STEP-9 trial, reported by Bliddal *et al* (213), demonstrated that the weekly administration of semaglutide at 2.4 mg for 68 weeks produced an additional 14.1-point improvement in the Western Ontario and McMaster Universities Osteoarthritis Index pain score compared with placebo in individuals with obesity and knee osteoarthritis.

Metformin, as a key activator of the AMPK signaling pathway, exerts multitarget metabolic regulatory effects and is considered to have substantial repurposing potential in metabolic OA. Available evidence indicates that its protective effects depend primarily on activation of the AMPKα1 isoform (215). In experimental models, metformin can inhibit chondrocyte senescence by regulating the inducible nitric oxide synthase/peroxynitrite/p53 signaling axis, while also promoting chondrocyte proliferation through the downregulation of microRNA-34a and the release of its inhibitory effect on SIRT1 (216,217). In addition, metformin can activate PINK1/Parkin-mediated mitophagy and promote the clearance of damaged mitochondria, while suppressing the expression of matrix-degrading enzymes from the MMP and ADAMTS families, thereby attenuating cartilage degeneration (218,219). In animal models, metformin has been shown to increase articular cartilage thickness and reduce cartilage damage, with more pronounced protective effects in obesity-related or high-fat diet-associated OA models (215,219). Clinical studies further suggest that the long-term use of metformin is associated with a lower rate of knee cartilage volume loss and may further reduce the risk of joint replacement when used in combination with cyclooxygenase-2 inhibitors (220,221). Taken together, preclinical evidence and observational cohort studies support the potential disease-modifying effects of metformin in metabolic OA; however, randomized OA-specific clinical trials are still required to confirm its therapeutic efficacy.

The potential value of sodium-glucose cotransporter 2 (SGLT2) inhibitors in OA intervention lies mainly in their anti-inflammatory and oxidative stress-modulating effects. Studies have shown that agents such as dapagliflozin can activate SIRT1 signaling and inhibit protein kinase R-like endoplasmic reticulum kinase/eukaryotic translation initiation factor 2α/C/EBP homologous protein-mediated endoplasmic reticulum stress, thereby reducing chondrocyte apoptosis and producing a chondroprotective phenotype characterized by increased type II collagen expression and decreased MMP13 and ADAMTS5 levels (222). In addition, these drugs can regulate the balance between autophagy and apoptosis through the activation of the AMPK pathway, upregulate autophagy-related proteins, such as Beclin 1 and ULK1, and suppress the expression of key Hedgehog pathway molecules, including sonic hedgehog and glioma-associated oncogene homolog 1 (223). *In vivo* studies have further indicated that SGLT2 inhibitors can reduce the serum levels of IL-1β, IL-6 and cartilage oligomeric matrix protein in animal models of OA, and improve joint space narrowing and osteophyte formation, without obvious toxicity within the reported dose range (222,223). Moreover, their combined use with methotrexate may further enhance anti-inflammatory and joint-protective effects, providing a novel potential therapeutic strategy for patients with OA accompanied by metabolic abnormalities (223). On the whole, the majority of current evidence for SGLT2 inhibitors in OA is derived from chondrocyte studies and animal models, and prospective clinical validation in patients with OA is still lacking.

### Circadian and lifestyle interventions

Melatonin, a crucial endogenous hormone that regulates circadian rhythm, has exhibited multidimensional protective potential in OA intervention. It has been demonstrated that melatonin can activate the PI3K/Akt-ERK-miR-185a signaling axis through the melatonin receptor 1, thereby suppressing synovial inflammation and angiogenesis and reducing the release of TNF-α, IL-8 and VEGF (66). In addition, melatonin can inhibit chondrocyte ferroptosis through the regulation of the NOX4/GRP78/GPX4 axis, reduce the accumulation of ROS and lipid peroxidation, and improve mitochondrial function (65). In terms of cartilage homeostasis, melatonin can regulate matrix metabolism through the SIRT1/NF-κB and TGF-β1/Smad2 pathway, promote the expression of Col2a1 and aggrecan, and suppress MMP activity (224). At the same time, melatonin can downregulate pain-related neuro mediators, such as nerve growth factor and calcitonin gene-related peptide, and has been shown to be associated with a reduced risk of joint replacement in patients with OA (72). In recent years, multiple delivery systems, such as melatonin-loaded poly (lactic-co-glycolic acid) nanoparticles functionalized with a collagen-binding peptide (MT@PLGA-COLBP), have been developed to improve the *in vivo* bioavailability of melatonin, and animal studies have demonstrated favorable long-term tolerability without evident organ toxicity (73,75). Therefore, although melatonin has exhibited consistent protective effects in experimental OA models and limited observational evidence in humans, its disease-modifying efficacy, optimal dosing, and target population remain to be established in prospective clinical trials.

Intermittent fasting is regarded as a potential non-pharmacological intervention through its effects on metabolic remodeling and inflammatory regulation. Intermittent fasting can induce a shift in metabolic substrate utilization and activate the AMPK/SIRT1 signaling axis, thereby improving mitochondrial function and alleviating insulin resistance (225). At the same time, intermittent fasting can reduce the release of pro-inflammatory mediators, such as TNF-α and IL-1β by suppressing NF-κB signaling, and can promote the clearance of damaged mitochondria through the activation of autophagy (225). Recent research further suggests that intermittent fasting can inhibit osteocyte-derived neuropeptide Y-mediated pro-inflammatory macrophage polarization and osteoclastogenesis, thereby weakening the pathological amplification process linking inflammation, bone destruction and cartilage injury (226). In addition, intermittent fasting may lower systemic inflammation through the gut-joint axis by reshaping gut microbial composition and promoting the production of SCFAs (227). In animal models and preliminary clinical studies, intermittent fasting has been reported to preserve cartilage structural integrity, suppress osteophyte formation, and improve pain and motor function. These metabolic and joint-protective effects may be further enhanced when intermittent fasting is combined with a high-protein diet (225,226). However, current evidence for intermittent fasting in OA is still based mainly on animal models and early clinical observations, and long-term human studies are warranted to determine its efficacy, safety, adherence and applicability across different OA phenotypes.

### Endocrine-metabolic phenotyping and mechanism-oriented therapeutic stratification

OA exhibits marked clinical and biological heterogeneity, which is also a key reason why conventional empirical treatments often produce variable outcomes. Classification based solely on the affected joint site or imaging findings often fails to capture the dominant pathogenic mechanisms underlying the disease (228). In recent years, increasing evidence has indicated that systemic metabolic dysregulation is a critical driver of OA, and that substantial differences in metabolic and endocrine phenotypes exist among patients. However, these factors have rarely been incorporated into traditional therapeutic decision-making frameworks, resulting in a lack of mechanism-oriented interventions (229). As the research paradigm of OA has gradually expanded from that of a local joint disorder to a systemic disease associated with disrupted whole-body metabolic homeostasis (14), the development of an endocrine-metabolic phenotyping framework may provide a basis for mechanism-oriented stratification and future therapeutic exploration.

From this perspective, patients with OA may be provisionally grouped according to dominant endocrine-metabolic features into two candidate phenotypic patterns, namely a hormone deficiency-dominant pattern and a metabolic syndrome-dominant pattern. The hormone deficiency-dominant pattern is primarily associated with abnormalities in endocrine regulation involving sex hormones, thyroid hormones, vitamin D, or PTH. Its pathological features are more inclined toward chondrocyte senescence, dysregulated subchondral bone remodeling, and impaired cartilage matrix synthesis, and it is commonly observed in postmenopausal women or individuals with endocrine disorders (230-232). By contrast, the metabolic syndrome-dominant pattern is driven mainly by systemic metabolic disturbances such as obesity, insulin resistance and dyslipidemia, with key pathological processes including chronic low-grade inflammation, abnormal adipokine signaling and oxidative stress (233-235). For example, signaling through the receptor for advanced glycation end products and related inflammatory responses can promote cartilage degradation, while metabolic abnormalities mediated by senescent immune cells may further aggravate damage to both the joint and musculoskeletal systems (236,237). In clinical practice, some patients may simultaneously exhibit hormone deficiency and metabolic abnormalities, thereby forming a mixed endocrine-metabolic pattern that requires more refined stratification and management strategies. This framework should be regarded as a hypothesis-generating model rather than a validated clinical taxonomy.

**Figure 3:**
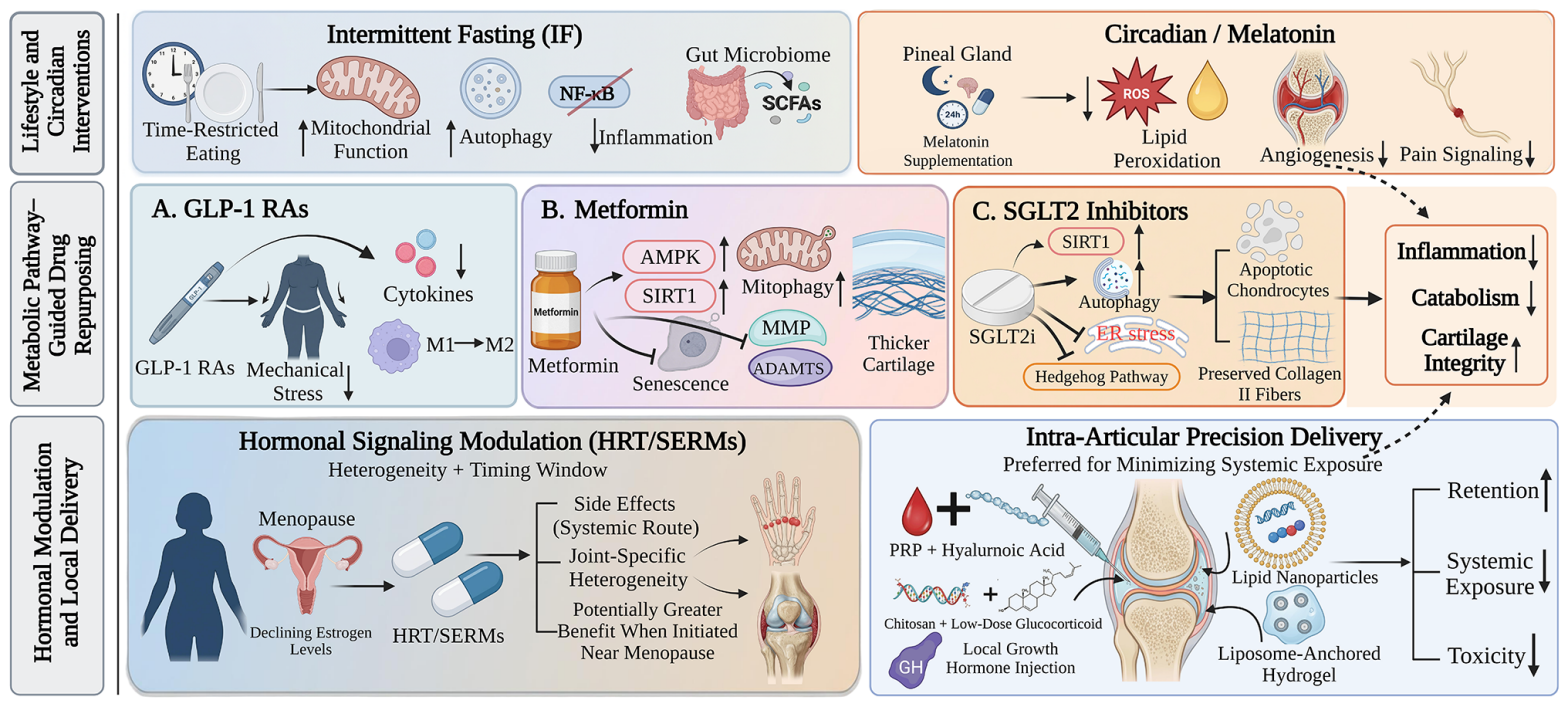
Therapeutic strategies targeting the endocrine-metabolic axis in OA. These interventions modulate inflammation, catabolic remodeling and cartilage integrity. Lifestyle and circadian approaches, such as time-restricted eating or intermittent fasting, enhance mitochondrial function and autophagy, suppress inflammation and increase SCFAs. Melatonin reduces ROS and lipid peroxidation, inhibits synovial inflammation and angiogenesis and alleviates pain signaling. Metabolic pathway-guided drug repurposing includes (A) glucagon-like peptide-1 receptor agonists, which lower mechanical load and pro-inflammatory cytokines and promote macrophage polarization from M1 to M2; (B) metformin, which activates AMPK and SIRT1, enhances mitophagy, suppresses senescence, and reduces matrix-degrading enzymes; and (C) SGLT2 inhibitors, which activate SIRT1-dependent autophagy, dampen endoplasmic reticulum stress and Hedgehog signaling, reduce chondrocyte apoptosis and preserve type II collagen. Hormone modulation, including hormone replacement therapy and selective estrogen receptor modulators, exhibits timing- and joint-dependent benefits, but carries systemic risks. Local delivery approaches, including PRP + HA, chitosan plus low-dose glucocorticoid, local growth hormone injection, lipid nanoparticles, and liposome-anchored hydrogels, aim to prolong joint retention and reduce systemic exposure. OA, osteoarthritis; IF, intermittent fasting; NF-κB, nuclear factor kappa B; SCFAs, short-chain fatty acids; ROS, reactive oxygen species; GLP-1, glucagon-like peptide-1; GLP-1 RAs, glucagon-like peptide-1 receptor agonists; AMPK, AMP-activated protein kinase; SIRT1, sirtuin 1; MMP, matrix metalloproteinase(s); ADAMTS, a disintegrin and metalloproteinase with thrombospondin motifs; SGLT2, sodium-glucose cotransporter 2; SGLT2i, sodium-glucose cotransporter 2 inhibitor; ER, endoplasmic reticulum; HRT, hormone replacement therapy; SERMs, selective estrogen receptor modulators; PRP, platelet-rich plasma. The figure was created in BioRender (https://BioRender.com/puoygvo).

With the development of multi-omics technologies and artificial intelligence (AI), molecular feature-based mechanism-oriented phenotyping and stratified therapeutic exploration may become increasingly feasible. Integrative multi-omics analyses can identify potential key molecules and regulatory networks through datasets such as transcriptomics and immunomics, including candidate targets, such as Jun proto-oncogene and VEGFA, as well as molecular biomarkers related to autophagy or inflammation, such as V-Erb-B2 avian erythroblastic leukemia viral oncogene homolog 2, thereby supporting the identification of candidate pathological patterns (238,239). At the same time, AI technologies can be used to automatically assess structural joint changes and assist therapeutic decision-making. For example, imaging analysis tools and clinical decision support systems may help predict disease progression or treatment response (240). In addition, federated learning provides a novel technical pathway for multicenter data integration and may improve model generalizability while preserving data privacy (241). Overall, the synergistic application of multi-omics and AI provides an essential technical foundation for mechanism-oriented OA phenotyping, stratified intervention design and treatment response assessment. A summary of the therapeutic strategies targeting the endocrine-metabolic axis in OA is illustrated in Fig. 3 and presented in Table III.

## 6. Conclusion and future perspectives

In recent years, the conceptual framework of OA has gradually shifted from that of a local degenerative disorder driven primarily by mechanical wear to that of a whole-joint disease jointly driven by local tissue injury and systemic endocrine-metabolic disequilibrium (7,242,243). As summarized in the present review, hormonal dysregulation, metabolic syndrome-related abnormalities, and immunometabolic stress do not act in isolation. Instead, they are tightly interconnected through key pathological processes including inflammatory amplification, disordered energy metabolism, mitochondrial dysfunction, PCD, and cellular senescence, and together they ultimately drive cartilage degeneration, synovial inflammation, and dysregulated subchondral bone remodeling (183,244,245). This evolving perspective not only broadens the systemic pathological landscape of OA, but also highlights its substantial biological heterogeneity. In particular, these provisional phenotypic patterns based on hormonal status and metabolic features, namely the hormone deficiency-dominant pattern and the metabolic syndrome-dominant pattern, may help explain, at the mechanistic level, the differences in disease progression, clinical phenotype, and therapeutic response observed among patients (230,246,247).

Although increasing evidence supports the role of the endocrine-metabolic axis in OA, several critical bottlenecks still hinder its clinical translation. First, the majority of existing models focus on a single mechanical insult, a single hormonal abnormality, or a single metabolic factor, and therefore remain insufficient to fully recapitulate the complex disease course of human OA, which is characterized by long-term accumulation and dynamic interaction of multiple pathogenic factors. Second, current clinical studies still provide an inadequate characterization of patient heterogeneity. This is particularly evident in the limited integration of evidence regarding sex differences, menopausal status, the synergistic effects of obesity and insulin resistance, and the identification of dominant mechanisms across candidate phenotypic patterns (21,127,248). In addition, endocrine-metabolic interventions, including GLP-1 receptor agonists, metformin, melatonin and intermittent fasting, have shown encouraging effects in preclinical studies, observational analyses or early clinical settings (213-215). Intra-articular local delivery systems, such as lipid nanoparticle- and hydrogel-based platforms, may also enhance joint retention and reduce systemic exposure (205-207). However, there remains a substantial gap between mechanistic association and confirmed clinical benefit, suggesting that future OA research should move beyond descriptive correlations and establish mechanistic frameworks that are testable, stratifiable and translatable.

Despite these advances, several limitations of the current available evidence should be acknowledged. The translational evidence for endocrine-metabolic interventions remains uneven. For example, melatonin has been shown to exert protective effects in experimental OA models and limited observational evidence in humans; however, long-term prospective human data are still insufficient, and its optimal dosage, treatment duration and target populations remain unclear (72-78). Similarly, evidence for hormonal interventions, including HRT and SERMs, remains heterogeneous and appears to be influenced by joint site, menopausal timing, formulation, treatment duration and long-term safety considerations, including cardiovascular and hormone-related risks (201-204). In addition, although multi-omics profiling, imaging analysis, and AI-based prediction models have identified promising molecular signatures and decision-support tools, few clinical studies have prospectively integrated endocrine indicators, metabolic status, imaging phenotypes, and multi-omics data to validate mechanism-based OA stratification (238-241,249-251). Finally, the hormone deficiency-dominant and metabolic syndrome-dominant patterns discussed in the present review should still be regarded as provisional phenotypic patterns rather than validated clinical categories. Future studies are required to define operational thresholds, predictive biomarkers, and treatment-response indicators before these patterns can be used to guide clinical decision-making (230,246,247).

Future OA research may no longer focus on identifying a single intervention target applicable to all patients, but rather on building an integrated classification framework based on hormonal status, metabolic characteristics and patterns of immunometabolic stress. On this basis, multi-omics technologies may further identify key molecular networks and biomarkers for different candidate phenotypic patterns (249,250), while AI, machine learning and clinical decision support tools may improve the feasibility of patient stratification, therapeutic response prediction, and dynamic monitoring (240,251). Overall, understanding OA through the lens of endocrine-metabolic interaction and advancing mechanism-oriented intervention through stratified classification may represent a key direction for future OA research and clinical management. It may also provide a useful reference for translational studies of other metabolism-related osteoarticular diseases.

## Figures and Tables

**Table I:** Key endocrine and metabolic mediators involved in the pathogenesis of osteoarthritis.

Mediator	Source	Target receptors	Major molecular effect	Clinical association	(Refs.)
Estrogen	Ovary	ERα, ERβ	Promotes chondrogenesis and type II collagen synthesis; inhibits MMP-related matrix degradation.	Protects joints; deficiency increases postmenopausal OA risk and severity.	(27,29,33)
Testosterone	Testis/ovary	Androgen receptor	Regulates osteoblast and osteoclast activity; maintains bone metabolism balance.	May protect joints; low levels associated with increased OA risk and pain.	(41,42, 46,47)
Thyroid hormones (T3/T4)	Thyroid gland	TRα	Induces chondrocyte hypertrophy; promotes ossification-related gene expression; accelerates matrix degradation.	Higher FT4 levels and altered thyroid-feedback indices have been associated with knee OA prevalence and severity; causal interpretation remains uncertain.	(52,54,56)
DIO2	Chondrocytes/ synovium	-	Converts T4 to bioactive T3 and promotes TRα-dependent hypertrophic signaling involving HIF-2α and RUNX2; promotes matrix degradation and mineralization.	Genetic variant promotes OA susceptibility and progression.	(58-60)
Melatonin	Pineal gland	MT1, MT2	Inhibits apoptosis and ferroptosis; reduces TNF-α, IL-8 and MMPs; regulates oxidative stress and inflammation.	Protects joints; may reduce pain and joint replacement risk.	(64,72,77)
Cortisol	Adrenal gland	Glucocorticoid receptor	Exerts anti-inflammatory effects via transcriptional regulation (effects are concentration-dependent).	Dysregulated rhythm (e.g., blunted awakening response) associated with increased pain.	(80-82)
PTH	Parathyroid gland	PTH1R	Regulates chondrocyte proliferation, matrix synthesis, and subchondral bone microstructure (context-dependent).	Genetically predicted higher PTH levels have been associated with lower hip/knee OA risk, while experimental PTH(1-34) may preserve cartilage and subchondral bone; protective effects context-dependent.	(84,86)
Vitamin D	Cutaneous synthesis and dietary intake	VDR	Regulates chondrocyte autophagy and metabolic pathways; maintains cartilage homeostasis.	Deficiency linked to joint pain and severity, but supplementation shows limited structural benefit.	(90,92,94)
IGF-1	Liver	IGF-1R	Regulates bone metabolism and chondrocyte function; maintains joint homeostasis.	Higher serum IGF-1 has been associated with increased hip and knee OA risk, but OA-specific therapeutic clinical evidence remains limited.	(95,151)
GHRH	Hypothalamus	GHRHR	Promotes chondrocyte proliferation and enhances extracellular matrix synthesis.	Provides protection against cartilage damage.	(96)
TGF-β	Bone tissue, kidneys, etc.	TGF-βR	Maintains cartilage homeostasis at low levels; aberrant activation exacerbates joint damage.	Dual role: protective at low levels, promotes damage if aberrantly activated.	(97,98)
VEGF	Bone tissue, vascular endothelium, etc.	VEGFR	Promotes angiogenesis and is involved in pain formation.	Promotes cartilage degeneration and contributes to OA pain.	(99)
Leptin	Adipose tissue	Ob-R	Activates JAK/STAT, NF-κB pathways; promotes pro-inflammatory factors and MMPs; inhibits cartilage matrix synthesis.	Promotes OA; positively associated with hand/knee OA risk, especially in females.	(21,23, 109)
Adiponectin	Adipose tissue	AdipoR1/2	Context-dependent: can activate AMPK (protective) or promote inflammation and affect apoptosis/autophagy.	Plays a bidirectional, context-dependent role in OA.	(110,111)
Visfatin	Adipose tissue	IR, LOX-1	Promotes expression of inflammatory cytokines and matrix-degrading enzymes.	Associated with OA severity.	(107,112)
Resistin	Adipose tissue	TLR4, TNFR1	Promotes expression of inflammatory cytokines and matrix-degrading enzymes.	Associated with OA severity.	(107,112)
Glucose	Diet/ metabolism	(Metabolic sensor)	Induces glycolysis, lactate accumulation, AGEs formation, ER stress, and oxidative stress in synovium and cartilage.	Hyperglycemia and poor glycemic control are linked to increased OA risk.	(113,114, 121)
Oxidized LDL	Lipid metabolism	LOX-1	Activates ERK1/2 and mTOR pathways; inhibits TFEB; induces oxidative stress, inflammation, and impaired autophagy.	Promotes cartilage degeneration, osteophyte formation, and synovitis.	(133,135, 137)
SCFAs	Gut microbiota	GPR41/43/ 109A; HDACs	Inhibits NF-κB and MAPK; reduces inflammatory factors and MMPs; improves chondrocyte autophagy.	Improves pain and function scores in knee OA patients; protects joints.	(142,147, 150)
LPS	Gut microbiota	TLR4	Enters circulation via disrupted gut barrier, induces systemic inflammation.	Promotes OA.	(138,139, 141)

AGEs, advanced glycation end products; AMPK, AMP-activated protein kinase; DIO2, type 2 deiodinase; ERα/β, estrogen receptor α/β; ERK1/2, extracellular signal-regulated kinase 1/2; FT4, free thyroxine; GHRH, growth hormone-releasing hormone; GHRHR, growth hormone-releasing hormone receptor; GPR41/43/109A, G protein-coupled receptors 41, 43, and 109A; HDACs, histone deacetylases; HIF-2α, hypoxia-inducible factor-2α; IGF-1, insulin-like growth factor 1; IGF-1R, insulin-like growth factor 1 receptor; INSR, insulin receptor; JAK/STAT, Janus kinase/signal transducer and activator of transcription; LOX-1, lectin-like oxidized low-density lipoprotein receptor-1; LPS, lipopolysaccharide; MAPK, mitogen-activated protein kinase; MMPs, matrix metalloproteinases; MT1/2, melatonin receptors 1 and 2; mTOR, mechanistic target of rapamycin; NF-κB, nuclear factor kappa B; OA, osteoarthritis; Ob-R, leptin receptor; PTH, parathyroid hormone; PTH1R, parathyroid hormone 1 receptor; RUNX2, runt-related transcription factor 2; SCFAs, short-chain fatty acids; T3, triiodothyronine; TFEB, transcription factor EB; TGF-β, transforming growth factor-β; TGF-βR, transforming growth factor-β receptor; TLR4, Toll-like receptor 4; TNF-α, tumor necrosis factor-α; TNFR1, tumor necrosis factor receptor 1; TRα, thyroid hormone receptor α; VDR, vitamin D receptor; VEGF, vascular endothelial growth factor; VEGFR, vascular endothelial growth factor receptor.

**Table II:** Selected programmed cell death pathways implicated in osteoarthritis.

Pathway	Main triggers in OA	Key molecules or axis	OA-relevant outcomes	Evidence status	(Refs.)
Pyroptosis	IL-1β, TNF-α, LPS, ATP, ROS and mechanical stress	NF-κB/MAPK-NLRP3-caspase-1-GSDMD axis; non-canonical caspase-4/5/11-GSDMD pathway	Promotes IL-1β and IL-18 release, inflammatory chondrocyte death, MMP and ADAMTS upregulation, synovitis, and cartilage matrix degradation	Cell, animal, and human OA-tissue evidence; no interventional clinical trials	(172-178)
Ferroptosis	Iron overload, lipid peroxidation, inflammatory stimulation, and mechanical stress	system xc^−^/GSH/GPX4 axis; SLC7A11, TFRC, ACSL4, LPCAT3, lipoxygenases, and ferritinophagy-related pathways	Induces lipid ROS accumulation, chondrocyte injury, MMP13 upregulation, type II collagen loss, and cartilage degeneration	Cell, animal, and human OA-tissue evidence; no interventional clinical trials	(172-174, 179-184)
Necroptosis	Cartilage trauma, inflammatory cytokines, oxidative stress and death receptor-related signaling	RIPK1-RIPK3-MLKL pathway; TRADD/RIPK1-related signaling	May contribute to chondrocyte loss, release of immunostimulatory cellular contents, inflammatory amplification, and cartilage destruction	Primarily cell and animal evidence	(172-174, 185,186)
Cuproptosis	Copper metabolic imbalance, hypoxic microenvironment, mitochondrial metabolic stress, and synovitis-related immune dysregulation	FDX1-dependent lipoylated TCA-cycle protein pathway; OA synovitis-related CRGs including FDX1, LIPT1, PDHA1, PDHB, and CDKN2A	May contribute to mitochondrial metabolic dysfunction, synovial inflammation, immune infiltration, and cartilage damage	Bioinformatic/single-cell plus limited mechanistic evidence	(174, 187-189)
PANoptosis	Combined inflammatory, oxidative, and danger-signal stimulation	Integrated pyroptosis, apoptosis, and necroptosis machinery; CASP8, CASP1, TLR3, IL-18, inflammasome- and RIPK-related components	May amplify inflammatory cell death and connect multiple PCD pathways during OA progression; functional OA-specific validation remains lacking	Exploratory in silico/ single-cell evidence only	(172,173, 190)

ACSL4, acyl-CoA synthetase long-chain family member 4; ADAMTS, a disintegrin and metalloproteinase with thrombospondin motifs; ATP, adenosine triphosphate; CDKN2A, cyclin-dependent kinase inhibitor 2A; CRGs, cuproptosis-related genes; FDX1, ferredoxin 1; GPX4, glutathione peroxidase 4; GSDMD, gasdermin D; GSH, glutathione; IL, interleukin; LIPT1, lipoyltransferase 1; LPS, lipopolysaccharide; LPCAT3, lysophosphatidylcholine acyltransferase 3; MAPK, mitogen-activated protein kinase; MLKL, mixed lineage kinase domain-like protein; MMP, matrix metalloproteinase; NF-κB, nuclear factor kappa B; NLRP3, NLR family pyrin domain-containing 3; OA, osteoarthritis; PANoptosis, pyroptosis, apoptosis, and necroptosis-related inflammatory cell death; PCD, programmed cell death; PDHA1, pyruvate dehydrogenase E1 subunit alpha 1; PDHB, pyruvate dehydrogenase E1 subunit beta; RIPK, receptor-interacting serine/threonine-protein kinase; ROS, reactive oxygen species; SLC7A11, solute carrier family 7 member 11; system xc−, cystine/glutamate antiporter system; TCA, tricarboxylic acid; TFRC, transferrin receptor; TLR3, Toll-like receptor 3; TNF-α, tumor necrosis factor-α; TRADD, tumor necrosis factor receptor type 1-associated death domain protein.

**Table III:** Therapeutic strategies targeting the endocrine-metabolic axis in osteoarthritis.

Intervention	Specific agent	Mechanism of action	Study design	Evidence level	Key findings	(Refs.)
HRT	Estrogen-based therapy	Upregulation of COL2A1; reduction of CTX-II and COMP levels	Feasibility randomized controlled trial in symptomatic hand OA; observational cohorts; meta-analysis of animal studies	Feasibility trial evidence; observational evidence; preclinical evidence	Early initiation may be associated with reduced hand OA risk, whereas some studies have linked HRT to increased knee OA or arthroplastyrisk; cardiovascular and hormone-related safety concerns limit long-term use	(36,201-204)
SERMs	Selective estrogen receptor modulators	Suppression of cartilage turnover	Systematic review and meta-analysis of menopausal animal models, primarily ovariectomized rodents	Preclinical evidence	Potential chondroprotective effects observed mainly in preclinical models; clinical OA evidence remains limited	(36)
Local delivery systems	PRP + HA	Synergistic anti-inflammatory and regenerative effects; improved intra articular symptom control	Clinical studies andmeta-analyses of intra-articular injectables	Clinical evidence	Improved analgesia and functional outcomes in selected clinicalstudies; regimen heterogeneity remains	(205-207)
Advanced local delivery platforms	Lipid nanoparticles; liposome-anchored hydrogels; chitosan-based formulations	Enhanced intra-articular retention and sustained drug release	Animal or early translational studies	Preclinical/early translational evidence	Improved intra-articular retention and sustained release in experimental models; clinical validation remains limited	(208,209)
GLP-1 RAs	Semaglutide; liraglutide	Reduced mechanical loading and systemic inflammation; NF-κB inhibition; M1→M2 macrophage polarization; MMP-3/13 downregulation	RCT (STEP 9) in participants with obesity and knee OA; observational cohort studies; preclinical studies	Randomized trial evidence in obesity-related knee OA; observational evidence; preclinical evidence	The STEP 9 RCT showed improved WOMAC pain in participants with obesity and knee OA. Observational data were associated with slower cartilage loss and lower arthroplasty risk. Generalization to non-obese or non-knee OA populations requires further validation	(210-214)
AMPK activators	Metformin	AMPKα1 activation; anti- senescence; PINK1/Parkin-mediated mitophagy; MMP/ADAMTS suppression	Cell and animal studies, including obesity/HFD- induced OA models; reviews summarizing limited observational and clinical evidence	Preclinical evidence; review-level clinical/ observational evidence	Cell and animal studies suggest reduced chondrocyte senescence, enhanced mitophagy, increased cartilage thickness, and reduced cartilage damage; reviews summarize limited clinical and observational evidence suggesting potential clinical relevance, but OA-specific randomized trials are still needed	(215-221)
SGLT2 inhibitors	Dapagliflozin	SIRT1 activation; inhibition of ER-stress-mediated chondrocyte apoptosis	Chondrocyte studies	Preclinical evidence	Preclinical evidence suggests reduced ER-stress-mediated chondrocyte apoptosis; efficacy in OA animal models and humans remains unvalidated	(222)
Circadian modulators	Melatonin	PI3K/Akt-ERK modulation; ferroptosis inhibition; SIRT1/NF-κB regulation	Animal and preclinical delivery studies; human cohort studies	Observational evidence; preclinical evidence	Suppressed synovial inflammation, angiogenesis, ferroptosis, and matrix degradation in experimental models; human cohort evidence suggested analgesic relevance and lower arthroplasty risk. Prospective efficacy and long-term safety data remain limited	(65,66,72, 73,75,224)
Lifestyle intervention	Intermittent fasting	AMPK/SIRT1 activation; NF-κB suppression; autophagy induction; gut microbiota remodeling via gut-joint axis	Animal studies and narrative review	Preclinical evidence	Preserved cartilage integrity and reduced osteophyte formation in animal models; effects on pain and function in human OA remain unestablished	(225-227)

ADAMTS, a disintegrin and metalloproteinase with thrombospondin motifs; Akt, protein kinase B; AMPK, AMP-activated protein kinase; COL2A1, collagen type II alpha 1 chain; COMP, cartilage oligomeric matrix protein; COX-2, cyclooxygenase-2; CTX-II, C-terminal telopeptide of type II collagen; ER stress, endoplasmic reticulum stress; ERK, extracellular signal-regulated kinase; GLP-1 RAs, glucagon-like peptide-1 receptor agonists; HA, hyaluronic acid; HFD, high-fat diet; HRT, hormone replacement therapy; MMP, matrix metalloproteinase; NF-κB, nuclear factor kappa B; OA, osteoarthritis; PI3K, phosphoinositide 3-kinase; PINK1, PTEN-induced putative kinase 1; PRP, platelet-rich plasma; RCT, randomized controlled trial; SERMs, selective estrogen receptor modulators; SGLT2, sodium-glucose cotransporter 2; SIRT1, sirtuin 1; WOMAC, Western Ontario and McMaster Universities Osteoarthritis Index.

## Data Availability

Not applicable.

## Abbreviations

OA: osteoarthritis
MMP: matrix metalloproteinase
TFQI: thyroid feedback quantile-based index
TNF-α: tumor necrosis factor-α
DIO2: type II deiodinase
PTH: parathyroid hormone
TGF-β: transforming growth factor-β
VEGF: vascular endothelial growth factor
AMPK: AMP-activated protein kinase
T2DM: type 2 diabetes mellitus
FLSs: fibroblast-like synoviocytes
ADAMTS: A disintegrin and metalloproteinase with thrombospondin motifs
NF-κB: nuclear factor κB
PI3K: phosphoinositide 3-kinase
mTOR: mechanistic target of rapamycin
ECM: extracellular matrix
SCFAs: short-chain fatty acids
ROS: reactive oxygen species
NLRP3: NLR family pyrin domain-containing 3
GLP-1 RAs: glucagon-like peptide-1 receptor agonists
SGLT2: sodium-glucose cotransporter 2
